# Faecal microbiota transplantation from patients with depression or healthy individuals into rats modulates mood-related behaviour

**DOI:** 10.1038/s41598-021-01248-9

**Published:** 2021-11-08

**Authors:** Julie Kristine Knudsen, Thomas Yssing Michaelsen, Caspar Bundgaard-Nielsen, René Ernst Nielsen, Simon Hjerrild, Peter Leutscher, Gregers Wegener, Suzette Sørensen

**Affiliations:** 1 Centre for Clinical Research, North Denmark Regional Hospital, Bispensgade 37, 9800 Hjørring, Denmark; 2 Department of Clinical Medicine, Aalborg University, Aalborg, Denmark; 3 Translational Neuropsychiatry Unit, Department of Clinical Medicine, Aarhus University, Aarhus, Denmark; 4 Department of Chemistry and Bioscience, Aalborg University, Aalborg, Denmark; 5 Department of Psychiatry, Aalborg University Hospital, Aalborg, Denmark; 6 Psychosis Research Unit, Aarhus University Hospital, Aarhus, Denmark; 7 Centre of Excellence for Pharmaceutical Sciences, North-West University, Potchefstroom, South Africa

**Keywords:** Behavioural methods, Biological models, Gene expression analysis, Genetic techniques, High-throughput screening

## Abstract

Differences in gut microbiota composition have been observed in patients with major depressive disorder (MDD) compared to healthy individuals. Here, we investigated if faecal microbiota transplantation (FMT) from patients with MDD into rats could induce a depressive-like phenotype. We performed FMT from patients with MDD (FMT-MDD) and healthy individuals (FMT-Healthy) into male Flinders Sensitive Line (FSL) and Flinders Resistant Line (FRL) rats and assessed depressive-like behaviour. No behavioural differences were observed in the FSL rats. In FRL rats, the FMT-Healthy group displayed significantly less depressive-like behaviour than the FMT-MDD group. However, there was no difference in behaviour between FMT-MDD FRL rats and negative controls, indicating that FMT-Healthy FRL rats received beneficial bacteria. We additionally found different taxa between the FMT-MDD and the FMT-Healthy FRL rats, which could be traced to the donors. Four taxa, three belonging to the family *Ruminococcaceae* and the genus *Lachnospira*, were significantly elevated in relative abundance in FMT-MDD rats, while the genus *Coprococcus* was depleted. In this study, the FMT-MDD group was different from the FMT-Healthy group based on behaviour and intestinal taxa.

## Introduction

An association between an altered gut microbiota composition and major depressive disorder (MDD) has been explored in several studies^1–18^. Changes in specific bacterial taxa have been investigated in patients with MDD in comparison to healthy control groups. Here, most studies found that the patient and control groups could be separated based on either α- or β-diversity, as well as an increased relative abundance of the genera *Eggerthella, Atopobium* and *Bifidobacteria*, and decreased *Faecalibacterium* in patients with MDD^19^. Differences in the structural composition of the gut microbiota have primarily been explored, but the functional effects of the bacteria present in the intestinal system have not been explored clinically. It is therefore difficult to deduce, if the observed changes in the microbiota are involved in a pathogenesis of MDD.

Different mechanisms potentially involved in the development of MDD have been hypothesized to be influenced by the gut microbiota. Intestinal bacteria may affect the brain by priming the immune system in the gut and subsequently in the body^20,21^, or by metabolizing intestinal tryptophan to serotonin, providing the host with up to 70% of the body’s total serotonin for neurotransmission^22^. On the other hand, the brain affects the gut by regulating gastrointestinal motility and secretion of hormones, in close cooperation with the enteric nervous system^23^. This bidirectional communication between the gut and the brain is termed the gut-brain axis^24^. This axis has been implicated in the pathogenesis and development of neuropsychiatric disorders, including MDD^24^. Different gut microbial species are known to produce metabolites that may result in mental and emotional changes^25^. Studies have supported this concept, as supplementation with probiotics, which produce metabolites such as the short chain-fatty acids (SCFAs), to patients with MDD resulted in a reduction of depressive symptoms^26,27^. It has furthermore been hypothesized that depressive-like behaviour in animal models arises from harmful bacteria-produced metabolites, such as lipopolysaccharides, entering the bloodstream^28–30^. Studies have observed reduced intestinal tight junction proteins in animals with gut microbiota alterations, which may increase barrier permeability, allowing the translocation of potentially harmful bacterial metabolites, which cannot normally pass this barrier^31,32^. This phenomenon, termed ‘leaky gut’, has been hypothesized to be involved in the pathogenesis and development of MDD^33,34^, as it is speculated that this allows transfer of metabolites leading to increased inflammation^35^.

Proof-of-concept studies have explored whether the gut microbiota of patients with MDD is involved in the pathogenesis and development of MDD. In four studies, faecal microbiota transplantation (FMT) was performed from patients with MDD into recipient animals^3,5,36,37^. Each study observed behavioural alteration in the animals receiving FMT from patients with MDD compared to control animals receiving FMT from healthy individuals. The alterations were plausibly linked to the transplantation of distinct bacteria with specific neuropathophysiological features. Although these studies found behavioural differences between animals receiving FMT from patients with MDD compared to healthy individuals, the applied animal models may have imposed behavioural differences prior to FMT. This necessitates the inclusion of additional control groups. Additionally, no previous study has attempted to reverse depressive-like behaviour by performing FMT from healthy individuals into an animal model of depression. To address this, the use of Flinders Sensitive Line (FSL) and Flinders Resistant Line (FRL) rats may be of scientific value. The FSL rat is a validated animal model of depression when behaviour is compared to the control FRL rat^38–40^. Previous studies of the FSL and FRL rat lines have found significantly different gut microbiota between the two groups^41^. Additionally, transplantation of FSL microbiota into FRL rats led to significantly elevated immobility in the forced swim test^41^. In these rats, it was furthermore found that depressive behaviours could be linked to dietary components manipulated by the gut microbiota^42^, and that probiotics could alter behaviour^42,43^.

The current work aimed to assess the behavioural changes in a rat model of depression upon transplantation of faeces from either antidepressant treatment-naïve patients with MDD or healthy individuals into both FSL and FRL rats. Moreover, we aimed to characterize the post-transplant gut microbiota in the recipient animals by 16S rRNA gene sequencing to elucidate if behavioural changes were associated with a shift in the gut community. Finally, we wanted to explore if there were any induced differences between the groups in tight junction protein expression in the caecum.

## Methods

### Human donors

Faecal samples were obtained from five antidepressant-naïve female patients recently diagnosed with first ever unipolar depression according to International Classification of Diseases, 10th Edition (ICD-10) criteria by a psychiatrist, as well as from five healthy female individuals. They were between 18 and 24 years of age, while the healthy individuals were between 23 and 30 years of age. The faecal material was obtained in continuation of a clinical study, which was conducted in accordance with the Helsinki Declaration. This study was approved by the Danish Ethical Committee (ID number: N-20170056). All participants gave informed consent to the use and application of their faecal samples in this study. The personal data collected in the project was registered in the processing activities of research in North Region of Denmark in compliance with EU GDPR article 30. Results from the clinical study will be published separately from the animal study.

### Animal recipients

Healthy 6–8-week-old (49.18 ± 4.86 days) male FSL and FRL rats were procured from the breading facility of the Translational Neuropsychiatry Unit, Department of Clinical Medicine, Aarhus University, Denmark. Animals were cohoused in pairs according to strain and birthmother in standard cages with access to ad libitum standard chow (#1324 Altromin, Brogaarden ApS, Lynge, Denmark) and water. The facility had a 12 h light/dark cycle (lights on at 8 A.M.), a temperature of 20 ± 2 °C and a relative humidity of 60 ± 5%. Animals were acclimatised to their new living conditions for 1 week before initiating the transplantation process. All animal experiments were conducted at the Translational Neuropsychiatry Unit, Department of Clinical Medicine, Aarhus University, approved by the European Union Council Directive on animal experimentation (ID Number: 2016-15-0201-01105) and conducted according to the Animal Research: Reporting of In Vivo Experiments (ARRIVE) guidelines^44^. All animal experiments were performed in accordance with guidelines on ethical conduct in animal research, including the 3 R’s (replacement, reduction and refinement)^45^. A total of 10 animals were used per group, as this sample size is necessary to determine a large enough effect size^46^.

### Faecal microbiota transplantation

Faecal samples from either the five patients with MDD or the five healthy individuals was pooled together for a total of 91 g. The characteristics of the participants are listed in Table 1.

**Table 1:** Demographic and clinical data on human participants.

Demographic and clinical data of donors	Patients with MDD	Healthy individuals	*p*-value
Age	21.6 (± 2.1)	26.4 (± 3.4)	0.03
Gender (% female)	100% (5/5)	100% (5/5)	1
BMI	24.0 (± 7.2)	23.0 (± 1.4)	0.69
Smoking (yes/no)	20% (1/5)	20% (1/5)	1
MDI score at inclusion	41,4 (± 2.7)	5.4 (± 2.3)	< 0.001
Gastrointestinal symptoms (yes/no)	40% (2/5)	0% (0/5)	0.14

Data displays the age, gender, BMI and smoking status of either patients with MDD or healthy individuals. Depressive severity was scored using the major depressive inventory. Data is displayed as mean ± SD. *BMI* Body mass index, *MDI* Major depressive inventory. *P*-values are displayed and are considered significant if *p* < 0.05.

Each participant supplied different amounts of faecal material. The pooled material from patients with MDD consisted of 16% (donor 1), 10% (donor 2), 58% (donor 3), 7% (donor 4) and 9% (donor 5). From healthy individuals, the pooled material consisted of 15% (donor 6), 23% (donor 7), 21% (donor 8), 28% (donor 9) and 13% (donor 10). Each pooled faecal sample was homogenised with 500 mL phosphate buffered saline and added 50 mL of glycerol to create a faecal solution. Rats were divided into nine groups (n = 10 per group). This sample size was chosen as this is the minimum requirement of animals to be able to determine significant differences between groups in the forced swim test^47,48^. This was also used as the primary outcome measure. Table 2 displays an overview of the nine groups of rats and their individual treatment.

**Table 2:** Overview of experimental animal groups.

Rat line	Sample size	Treatment
FSL	n = 10	FMT from patients with MDD (FMT-MDD)
	n = 10	FMT from healthy individuals (FMT-Healthy)
	n = 10	Control with autotransplantation of own faeces (CON-Auto)
	n = 10	Control with oral gavage of demineralised water (CON-H2O)
FRL	n = 10	FMT from patients with MDD (FMT-MDD)
	n = 10	FMT from healthy individuals (FMT-Healthy)
	n = 10	FMT from patients with MDD combined with sertraline (FMT-MDD-Ser)
	n = 10	Control with autotransplantation of own faeces (CON-Auto)
	n = 10	Control with oral gavage of demineralised water (CON-H2O)

FSL (n = 40) and FRL (n = 50) rats were used. Animal groups (n = 10 per group) received either faecal microbiota transplantations (FMT) from patients with MDD (FMT-MDD), FMT from healthy individuals (FMT-Healthy), FMT with their own faeces (CON-Auto) or water by oral gavage (CON-H2O). Additionally, one group of FRL rats received FMT from patients with MDD combined with simultaneous treatment with sertraline (FMT-MDD-Ser).

Five groups of rats received faecal transplants from human donors as follows: Patients with MDD (FMT-MDD, FSL n = 10 and FRL n = 10). Patients with MDD, then subsequently administered sertraline hydrochloride (Carbosynth) at a dose of 16.7 mg/kg/day at a minimum of three hours after FMT by oral gavage (FMT-MDD-Ser, FRL n = 10). Healthy individuals (FMT-Healthy, FSL n = 10 and FRL n = 10). Sertraline hydrochloride was administered chronically at a dose of 16.7 mg/kg/day, as the FSL rat only responds to chronic antidepressant treatment^49–51^ and this dose resembles clinically relevant dosages^52^.

Four additional groups did not receive FMT from humans and served as controls. In two of the four groups, faecal samples were collected directly from the co-housed animals, pooled and homogenized to create an animal faecal solution identical in concentration to the human faecal solutions, which was administered back into the rats (CON-AUTO, FSL n = 10, FRL n = 10). The other control groups received demineralised water in a volume identical to the volumes administered during the FMT procedures (CON-H2O, FSL n = 10, FRL n = 10).

FMT was performed thrice weekly for a 3 week period by oral gavage in a volume corresponding to the recommended guidelines by the European Consensus Conference on FMT in clinical practice^53^. This resulted in a faecal sample dosage of 0.42 g/kg with a solution of 0.9 mL administered per FMT. The CON-H2O group was administered 0.9 mL demineralised water thrice weekly for three weeks. Animals were excluded from the study if they stopped gaining weight, or lost weight, during the three weeks FMT was performed.

### Behavioural assessment of animals

Behavioural tests were performed two days after the last FMT in the order *open field test—rotarod test—training-session forced swim test*. The *testing-session forced swim test*, from which depressive-like behaviour was evaluated, was performed on the following day. Animals were moved into the behavioural facility one hour before initiating the experiments to acclimatise in a room with a red light of 35–45 lx. All test procedures were performed by the same experimenter specialized in animal behavioural assessment.

#### Open field test

The open-field test was performed under white light of 35–45 lx as previously described to measure locomotor activity^54,55^. Movement trajectory was recorded for ten minutes using a camera positioned directly above the open field. Locomotor activity was analysed using the Noldus Ethovision XT v. 14 software (Wageningen, Netherlands). The open field test was used as a control to ensure that alterations in struggling, swimming or immobile behaviour in the forced swim test was not due to differences between groups in overall locomotor activity.

#### Rotarod test

Animals were transferred directly from the open field test room to the rotarod test room with a dimmer light of 10–15 lx. Animals were individually placed on a spinning cylinder (Ugo Basile) specifically designed to rotate at increasingly higher speeds. The time-to-exhaustion was determined as the time passed until the rat fell off the rotating cylinder and measured to ensure that immobility in the forced swim test was not due to animal variations in energy output. The rotarod test was included as a control to ensure that differences in struggling, swimming or immobile behaviour in the forced swim test was not due to differences in endurance between groups.

#### Forced swim test

A modified version of the forced swim test was employed. The modification included a six-minute forced swim training-session, 24 h prior to the six-minute test-session^47,48^. The pre-swim training session was reduced to six minutes, so as to reduce the stress imposed on the animal, and as this length of time has previously been found to be as effective as a 15-min training session (unpublished data). Videos of the test sessions were then randomized and scored by an observer. Immobility, swimming and struggling behaviour were scored as the dominant behaviour displayed every five seconds and interpreted as passive coping (depressive-like), swimming, or active escape (antidepressant) behaviour. The first five minutes of the test-session videos were analysed. One FRL rat from the FMT-Healthy group was not fully submerged into the water during placement in the cylinder and was thus excluded from the behavioural analysis.

### Gene expression analysis

Gut tissue was collected from the animals to evaluate the expression of tight junction proteins in the caecum. Animals were euthanised by decapitation, the intestines macrodissected, and the base of the caecum was separated and approx.. 200 mg was placed in RNAlater (Thermo Fischer) before long-term storage at − 80 °C. Total RNA was extracted using 30 mg of tissue with the Fibrous Tissue RNeasy Mini kit (QIAGEN) according to manufacturer’s recommendations, including an additional DNAse step. RNA yield was measured using the NanoDrop (ThermoFischer). Integrity was assessed using the Qubit RNA IQ kit (ThermoFischer) and samples were only used if the RNA integrity number was above 7. Gene expression levels of rat *Ocln* (occludin), *Cldn3* (claudin-3) and *Gapdh* (GAPDH) were measured using a two-step qRT-PCR approach. First, cDNA was produced using the Affinity Script qPCR cDNA Synthesis kit (Agilent) according to manufacturer’s instructions followed by qPCR with the Brilliant III SYBR Green qPCR Master Mix (Agilent) according to manufacturer´s recommendations. All samples were analyzed in triplicates using 20 ng of input cDNA with the following cycling conditions: 95 °C for 10 min, with 40 cycles of 95 °C for 1 min, 58 °C for 30 s (for *ocln* and *cldn3*) or 60 °C for 30 s (for *gapdh*), and 72 °C for 30 s. Primers were designed using the NCBI primer blast resource (https://www.ncbi.nlm.nih.gov/tools/primer-blast/) targeting all variants of rat *Ocln*, *Cldn3* and *Gapdh*. Details regarding primer sequences, sequence accession number and product length is displayed in Table 3.

**Table 3:** Overview of primers.

Gene target	Sequence accenssion number	Forward primer	Reverse primer	Product size
Ocln	NM_031329	5′-AACCGACTACACGACAGGTG-3′	5′-AGCCATGTACTCTTCGCTCTC-3′	96
Cldn3	NM_031700	5′-GAATGGACAAAGACACCTCGC-3′	5′-CCACTATGAGCCTTCTGGCTG-3′	129
Gapdh	NM_017008 XM_216453	5′-AGTGCCAGCCTCGTCTCATA-3′	5′-GGTAACCAGGCGTCCGATAC-3′	77

Targets include the genes for Occludin (*Ocln*), Claudin-3 (*Cldn3*), and Glyceraldehyde 3-phosphate dehydrogenase (GAPDH, *Gapdh*).

Relative expression levels of *Ocln* and *Cldn3* were determined by the ΔΔCt method against *Gapdh* expression., using the mean of *Gapdh* expression from the CON-H2O FRL rats as control expression.

### 16S rRNA gene sequencing

Faecal samples collected from rats before transplantation (pre-FMT) and after transplantation (post-FMT) were stored for a maximum of three hours on dry ice before long-term storage at − 80 °C. DNA was extracted from the human and rat faecal samples using the QIAamp PowerFecal Kit (Qiagen) as previously described^56^. DNA concentration was determined by a Qubit 4 fluorometer (Thermo Fischer). Gene sequencing of the 16S rRNA gene was performed by DNAsense, Denmark, on a MiSeq (Illumina)^57^ with primers 515F/806R targeting the hypervariable V4 region^58,59^ as previously described^60^. To ensure the quality of sequencing, a 20% PhiX control library (Illumina, USA), a negative control (nuclease-free water), and a positive control (anaerobic digester system sample) were added before sequencing.

### Bioinformatics and statistics

Data analysis was performed in R v. 3.6.0 through Rstudio v. 1.1.383 (http://www.rstudio.com) primarily using the packages ampvis2, tidyverse, and vegan. Forward and reverse reads were trimmed using Trimmomatic v0.32^61^ and merged using FLASH v1.2.7^62^. The data was then processed using the USEARCH amplicon workflow^63^, implemented in QIIME^64^. Operational taxnomic units (OTUs) were clustered to 97% sequence identity and assigned taxonomy using the MiDAS database v.2.1.3^65^. Samples were rarefied to the lowest observed sequencing depth for richness and diversity estimates. Community richness was calculated using the observed number of unique OTUs and diversity was calculated using the Shannon index. β-diversity was examined using principal component analysis (PCA) on Hellinger transformed OTU abundances. To assess the statistical significance of the groupings in PCA, averages of each grouping levels were fitted to the ordination using the envfit function from the vegan package. To analyse differential abundance of OTUs between groups from pre-FMT to post-FMT samples, the DESeq2 v.1.20.0 workflow was used^66^. Distribution and variance of continuous data were analysed using Shapiro-Wilk's test and Bartlett’s test, respectively. Data was tested using either ANOVA followed by Tukeys post hoc test, or Kruskall-Wallis test followed by Dunn’s post hoc test depending on normality and variance. For statistical comparison of pre-FMT and post-FMT results, the Wilcoxon rank sum test was used. The Benjamin-Hochberg *p*-value correction was used to address multiple testing^67^. An adjusted *p*-value < 0.05 was considered statistically significant. The bacterial sequencing data is freely available through the NCBI Sequence Read Archive under the ID number PRJNA741104. Data regarding behavior or gene expression of tight junction proteins is available upon request.

## Results

### Faecal microbiota transplantation from humans into FRL rats can modulate the behavioural phenotype

**Figure 1:**
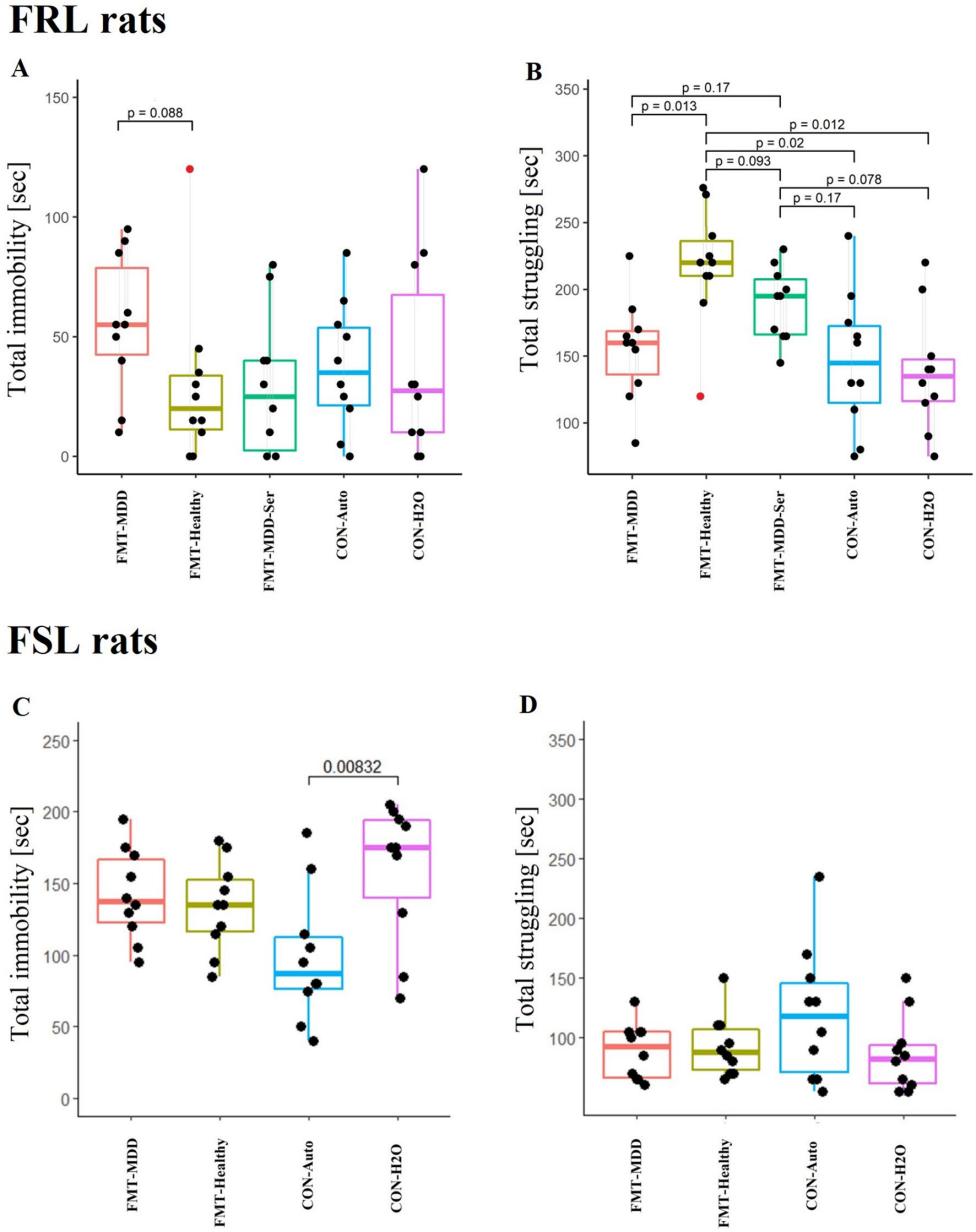
Behavioural analysis of the forced swim test of FSL and FRL rats. Total immobility time (sec) (**A**,**C**) and total struggling time (sec) (**B**,**D**) of rats from each group of FRL (**A**,**B**) or FSL (**C**,**D**) rats based on recordings of the first 5 min. *P*-values were included in the figure if they were below 0.2. Grey lines represent cohoused animals in the FRL group. The red dot represents the rat that was removed from the analysis as mentioned in the Methods section. FMT-MDD: Faecal microbiota transplantation from patients with MDD into rats. FMT-Healthy: Faecal microbiota transplantation from healthy individuals into rats. FMT-MDD-Ser: Faecal microbiota transplantation from patients with MDD into rats combined with treatment with sertraline. CON-Auto: Rats receiving auto-transplantations. CON-H2O: Rats receiving demineralised water.

The forced swim test was applied to evaluate whether FMT from patients with MDD could induce depressive-like behaviour in rats. In the FRL rats, there was a tendency towards higher immobility (*p* = 0.088) and significantly less struggling (*p* = 0.013) (Fig. 1A,B, respectively) in the FMT-MDD group compared to the FMT-Healthy group. Administration of sertraline resulted in a behavioural phenotype lying between that displayed by the FMT-MDD and FMT-Healthy groups. However, the control groups, CON-AUTO and CON-H2O showed comparable immobility and struggling times to the FMT-MDD groups. This could imply that the transfer of faeces from healthy individuals imposed a beneficial effect on inherent depressive-like behaviour instead of an adverse impact from the faeces of patients with MDD.

In contrast to the FRL rats, FSL rats did not display altered behaviour regardless of treatment (Fig. 1C,D). To ensure that the behaviour observed during the forced swim test was not caused by differences in locomotor activity or time-to-exhaustion, the animals also underwent the open field test and the rotarod test (Supplementary Fig. 1A,B, respectively). There was no difference between the five FRL groups in total distance travelled in the open field test. In the Rotarod test there was like-wise no difference between the groups, except for a statistical difference between the FMT-Healthy and the CON-AUTO groups, with the CON-Auto group spending significantly more time on the Rotarod (*p* = 0.015).

### Donor bacteria can be observed in faecal samples of recipient rats

**Figure 2:**
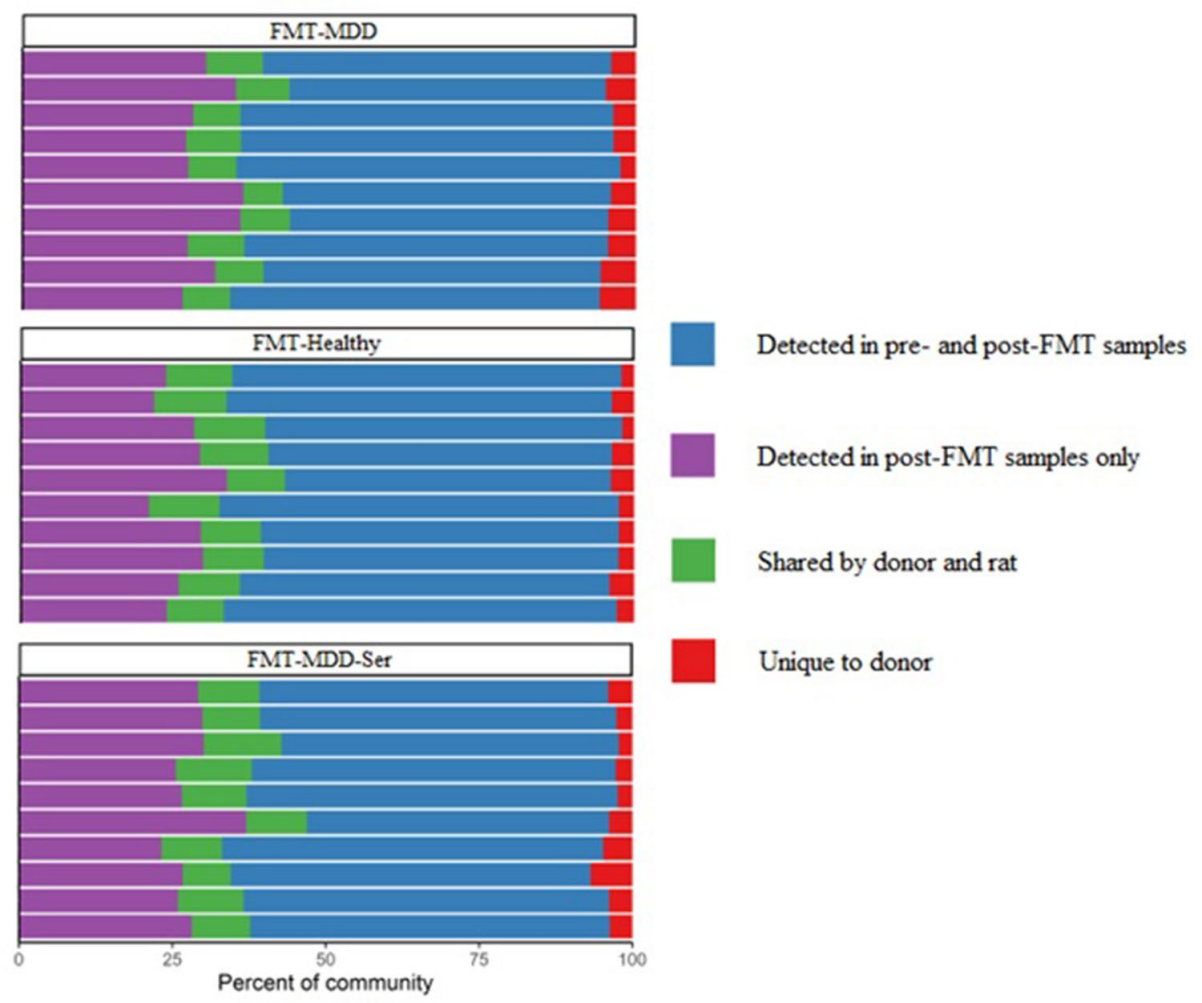
Stacked bar plots of relative changes in gut microbiota composition of FRL rats. Each bar represents all the OTUs observed across samples from one rat. OTUs in blue were detected in both pre-and post-FMT samples, while OTUs in purple were novel in the rat and not detected in the donor material. The green OTUs were common in both rats and humans, while the red percentage was unique to the human donor material only. FMT-MDD: Faecal microbiota transplantation from patients with MDD into rats. FMT-Healthy: Faecal microbiota transplantation from healthy individuals into rats. FMT-MDD-Ser: Faecal microbiota transplantation from patients with MDD into rats combined with treatment with sertraline.

We assessed whether the bacterial composition of the recipient rats was affected by the FMT. For this purpose, 16S rRNA gene sequencing was performed on faecal samples collected before and after transplantation. Human donor bacteria could be detected in FRL rats after transplantation, but only accounted for approx. 9.7 ± 1.5 SD % of the overall gut microbiota composition (Fig. 2).

This contribution was not sufficient to induce changes in α-diversity between pre- and post-treatment groups (Supplementary Fig. 2). Analyses of β-diversity, on the other hand, showed that post-treatment groups were significantly different from each other (Supplementary Fig. 3). The data, however, also showed that the FMT-MDD rats differed in gut microbiota composition compared to FMT-Healthy rats prior to the FMT procedure.

**Figure 3:**
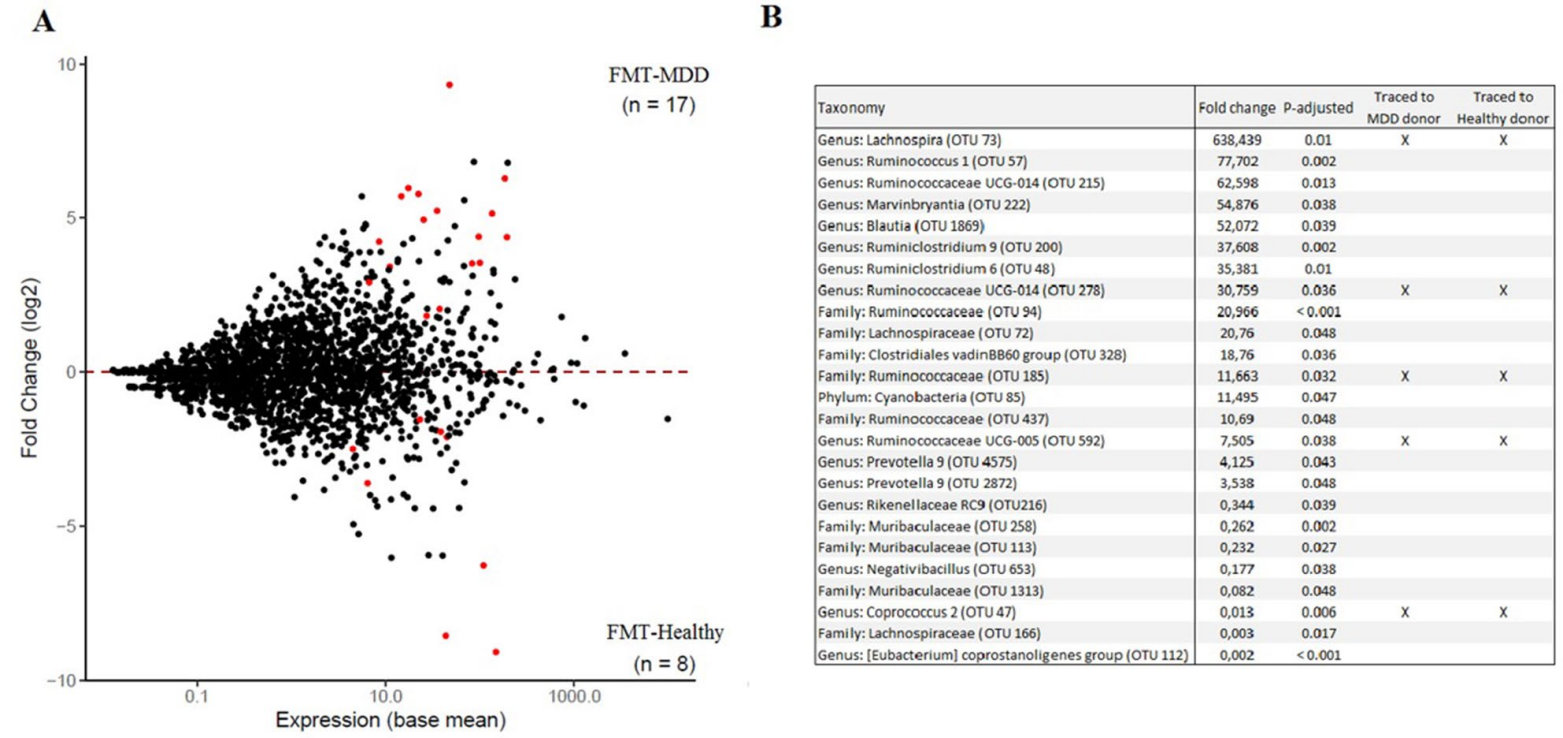
MA plot presenting the fold change in OTU expression between pre-FMT and post-FMT samples collected from FRL rats. (**A**) OTU expression in rats receiving faecal microbiota transplantation (FMT) from patients with MDD (FMT-MDD) compared to rats receiving from healthy individuals (FMT-Healthy). Of all the OTUs, seventeen were elevated in FMT-MDD samples, while eight were depleted compared to FMT-Healthy samples. (**B**) The twenty-five OTUs significantly different between FMT-MDD animals and FMT-Healthy animals. Five of these OTUs, marked by “X”, could be traced to the human donor material. The fold change is expressed as the change in relative abundance of bacteria in the FMT-MDD group compared to the FMT-Healthy group.

In addition, we assessed whether individual bacterial phylotypes were significantly different between the groups. In total, seventeen taxa were increased in relative abundance in FMT-MDD rats compared to FMT-healthy rats, and eight were decreased (Fig. 3A). Of these, five taxa could be traced to the donor material, but with significantly different fold changes depending on the donor group (Fig. 3B). The genus *Lachnospira* and three genera belonging to the *Ruminococcaceae* family were overrepresented in relative abundance in FMT-MDD rats, while the genus *Coprococcus* was underrepresented.

### FMT from humans induced altered intestinal tight junction gene expression

**Figure 4:**
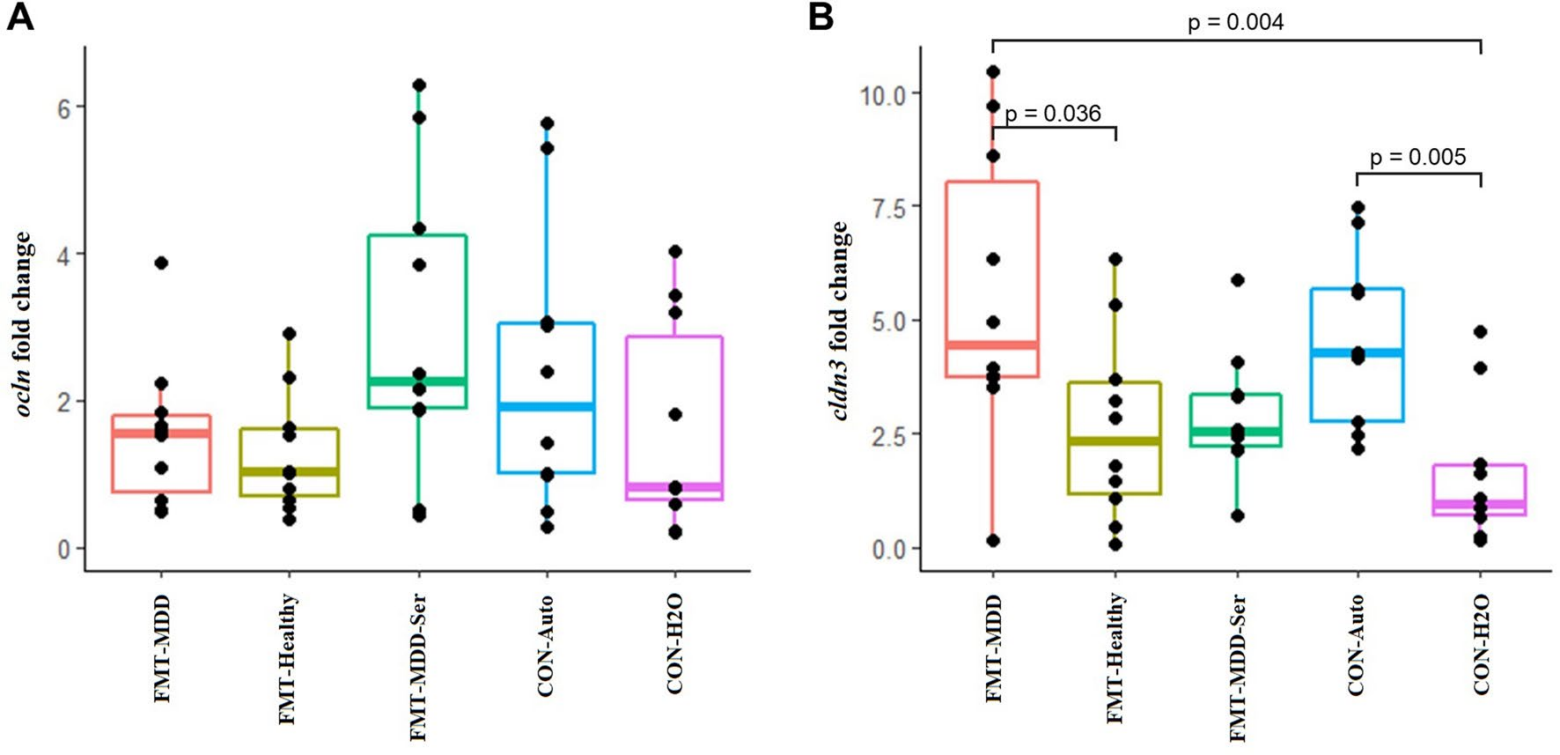
Relative mRNA expression of tight junction protein-encoding genes in the caecum of FRL rats. (**A**) Relative gene expression of ocln in rats. (**B**) Relative gene expression of *cldn3* in rats. Gene expression was normalized against gene expression of *Gapdh* using the ΔΔCt method. FMT-MDD: Faecal microbiota transplantation from patients with MDD into rats. FMT-Healthy: Faecal microbiota transplantation from healthy individuals into rats. FMT-MDD-Ser: Faecal microbiota transplantation from patients with MDD into rats combined with treatment with sertraline. CON-Auto: Rats receiving auto-transplantations. CON-H2O: Rats receiving demineralised water.

Induction of behavioural differences after transplantation with foreign bacteria may be associated with changes in the local intestinal gene expression of tight junction proteins^68^. To investigate this, we measured mRNA expression levels of the tight junction protein-coding genes, *ocln* and *cldn3* (Fig. 4). Surprisingly, there was a significantly higher relative gene expression of *cldn3* in FMT-MDD rats compared to FMT-Healthy and CON-H2O, indicating an upregulation of one of the tight junction proteins. Furthermore, there was also observed increased *cldn3* expression in the CON-Auto group compared to the CON-H2O, suggesting that this group resembles the FMT-MDD group in intestinal expression of this tight junction protein. Expression levels of *ocln* were not observed to be different between groups.

## Discussion

We explored if a depressive-like phenotype was induced in rats by FMT from treatment-naïve patients with MDD compared to FMT from healthy individuals. Here, we observed that the FMT-MDD group displayed depressive-like behaviour compared to the FMT-Healthy group, but not compared to the control groups CON-Auto, or CON-H2O. While there were no significant changes in α- or β-diversity from pre-FMT to post-FMT, individual phylotypes were observed to be significantly different between FMT-MDD and FMT-Healthy, five of which could be traced to the donor material.

### Behavioural phenotypes observed in this study are similar to previous reports despite animal model differences

The FMT-MDD group tended towards higher immobility and significantly less struggling in the forced swim test compared to the FMT-Healthy animals. This indicated that the microbiota from patients with MDD may be able to induce a depressive-like phenotype, as lack of struggling and increased immobility are interpreted as a display of a depressive phenotype^47^. Furthermore, in our study, treatment with sertraline simultaneous with FMT from patients led to a trend towards a less depressive phenotype. However, the FMT-MDD group did not display less struggling than the control groups, suggesting that FMT from healthy individuals rather has an antidepressant-like effect, than FMT from patients with MDD resulting in a depressive-like phenotype. Furthermore, the CON-Auto group resembled the FMT-MDD group more than the FMT-Healthy group, suggesting that reintroduction of bacteria into the animal results in a similar behavioural phenotype as the FMT-MDD group. As a result, it was not possible to establish an association between the gut microbiota of patients with MDD and induced depressive-like behavior in FMT-recipient rats, as this is not evident from the behavior in comparison to the CON-Auto or the CON-H2O groups. Although the FMT-Healthy does appear to decrease depressive-like behavior in the FRL rat, this was not observed in the FSL rat. This suggests that should FMT from healthy individuals into FRL rats induce an antidepressant phenotype, this cannot be replicated in a genetic animal model of depression like the FSL rat.

Although previous studies did observe a depressive-like phenotype when comparing animals receiving FMT from patients with MDD to animals receiving FMT from healthy individuals^3,5,36^, these studies did not involve control groups which did not receive FMT with human material. It can therefore be difficult to discern if the behavioural output from the animals arose from the FMT of foreign bacteria, or their inherent, natural behaviour. Additionally, previous studies used patients with MDD receiveing active pharmacological treatment, whereby it can be suggested that the gut microbiota composition may have partly shifted away from a ‘depressogenic’ composition. On the other hand, one study has included an additional control group and contrarily to our study, they observed depressive-like behaviour in their animals receiving FMT from patients with MDD compared to the non-intervention control rat^37^. This may be due to strain differences (Sprague–Dawley rats in their study, versus FSL/FRL rats in ours). It may also be due to differences in the animal model, as their rats were germ-free prior to transplantation, while our model did not include pre-treatment. However, all studies with FMT from patients with MDD compared to FMT from healthy individuals did observe significant behavioural differences between these two groups, despite apparent strain and animal model variations.

### Bacterial metabolites may facilitate behavioural differences

Five OTUs were observed to be significantly altered in recipient FMT-MDD and FMT-Healthy animals, which could be traced to the donor material; three from the family *Ruminococcaceae* and one from the genus *Lachnospira* were enriched in FMT-MDD animals, while the genus *Coprococcus* was depleted, compared to the FMT-Healthy group. *Lachnospira* has not previously been reported enriched in neither patients diagnosed with MDD^1–18^, nor in the previously performed studies of FMT^3,5,36,37^, but the family *Lachnospiraceae* has been observed significantly altered in both clinical studies^1–3,7,9,10,16,17,27^ and in one of the FMT studies^3^. *Lachnospira* has furthermore been observed in serotonin transporter knockout rats with genetic serotonin transporter knockout receiving early life stress^69^, a validated animal model of depression^70^. This suggests that *Lachnospira* may promote behavioural changes through interactions with neurotransmitter systems, which is also one of the primary interactions in the gut-brain axis^24^.

Low relative abundance of *Coprococcus* has previously been recognized in clinical assessments of the gut microbiota in patients with MDD^3,10,12,18^, and in mice subjected to social defeat stress, an animal model of depression^71^. *Coprococcus* is a prominent producer of SCFAs^72^, metabolites catalysed from undigestible, complex fibres. The severity of MDD has been positively associated with plasma SFCA concentration^73^, and faecal SCFA content is reduced in clinical assessments of gut microbiota in patients with MDD^2,3^. This suggests that increased *Coprococcus* in the FMT-Healthy group may be the mediator of the antidepressant-like behaviour observed compared to FMT-MDD.

In this study, we were able to induce an antidepressant effect of FMT from healthy individuals compared to FMT from patients with MDD into FRL rats. Furthermore, this can be associated to the intestinal bacteria observed in the rat after FMT, which could be traced to the donor material. Furthermore, future studies should include additional control groups, as was done in this study, to be able to discern that FMT from patients with MDD is depressogenic or if rather FMT from healthy individuals can rescue an underlying depressive phenotype.

### Limitations and strengths

The primary limitation in our study is the presence of a gut microbiota in recipient rats prior to FMT, reducing the colonization success of the donor material. Furthermore, the faecal material used for transplantation was pooled from five different individuals. In the bioinformatical processing, we used OTUs instead of amplicon sequence variants, which limits detection of bacteria at species level.

The strengths of this study include the addition of several control groups to evaluate the effect of FMT contrary to the animal’s natural behaviour. Using animals with inherent gut microbiota also has great translational value, as it mimics the human gastrointestinal system more closely. The donor material was collected from treatment-naïve patients with MDD, which has not been done previously. Additionally, we assessed the effect of antidepressant treatment on the behavioural effect imposed by FMT, which has not been explored in previous publications.

## Supplementary Information

Supplementary Figure 1.

Supplementary Figure 2.

Supplementary Figure 3.

Supplementary Legends.
