# Supplementary figures and images for: Faecal microbiota transplantation from patients with depression or healthy individuals into rats modulates mood-related behaviour
Source: Sci Rep. 2021 Nov 8;11:21869. doi: 10.1038/s41598-021-01248-9 (PMC8575883; doi:10.1038/s41598-021-01248-9)

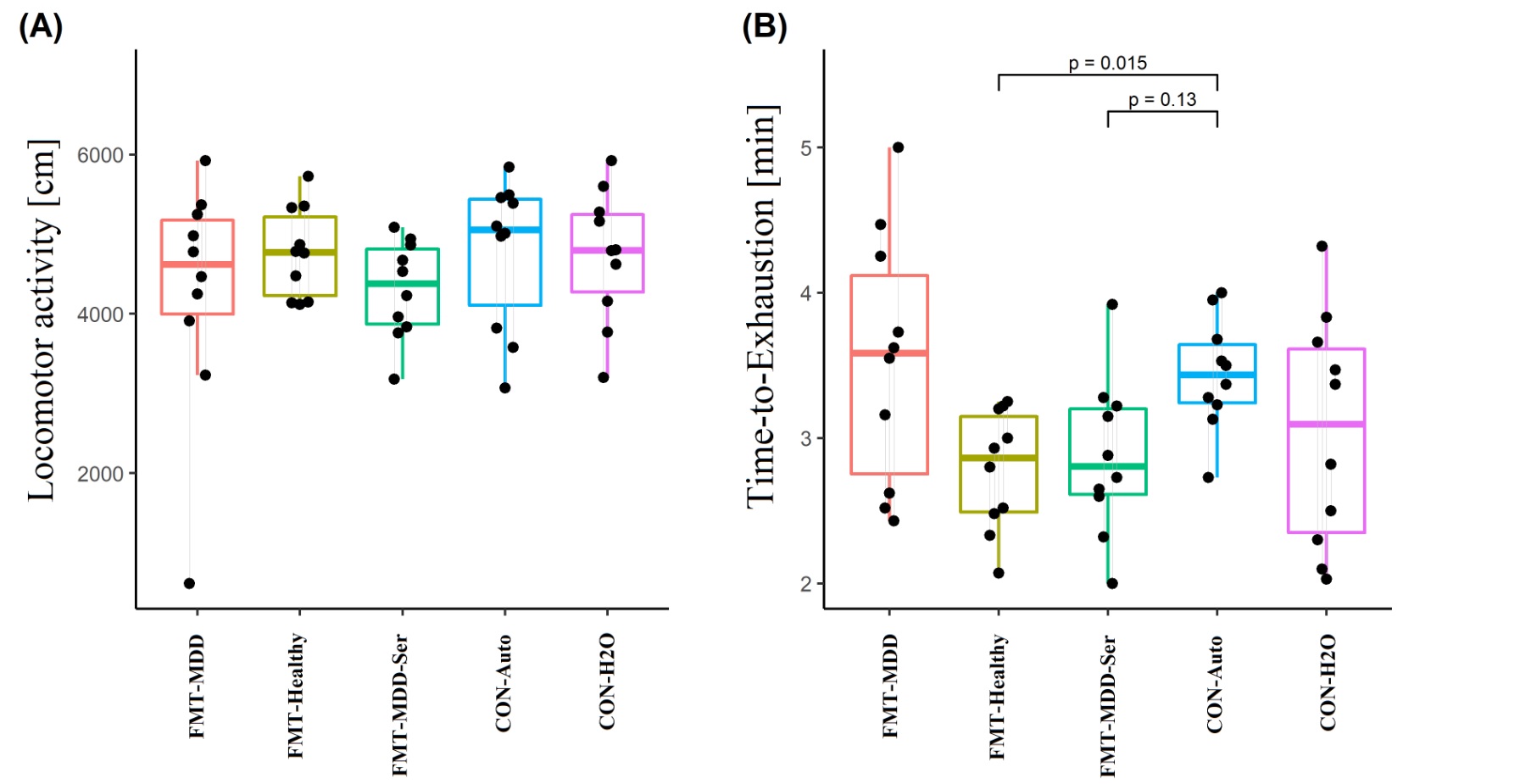

Supplement: Supplementary file 1 — Supplementary Figure 1. [file 41598_2021_1248_MOESM1_ESM.jpg]

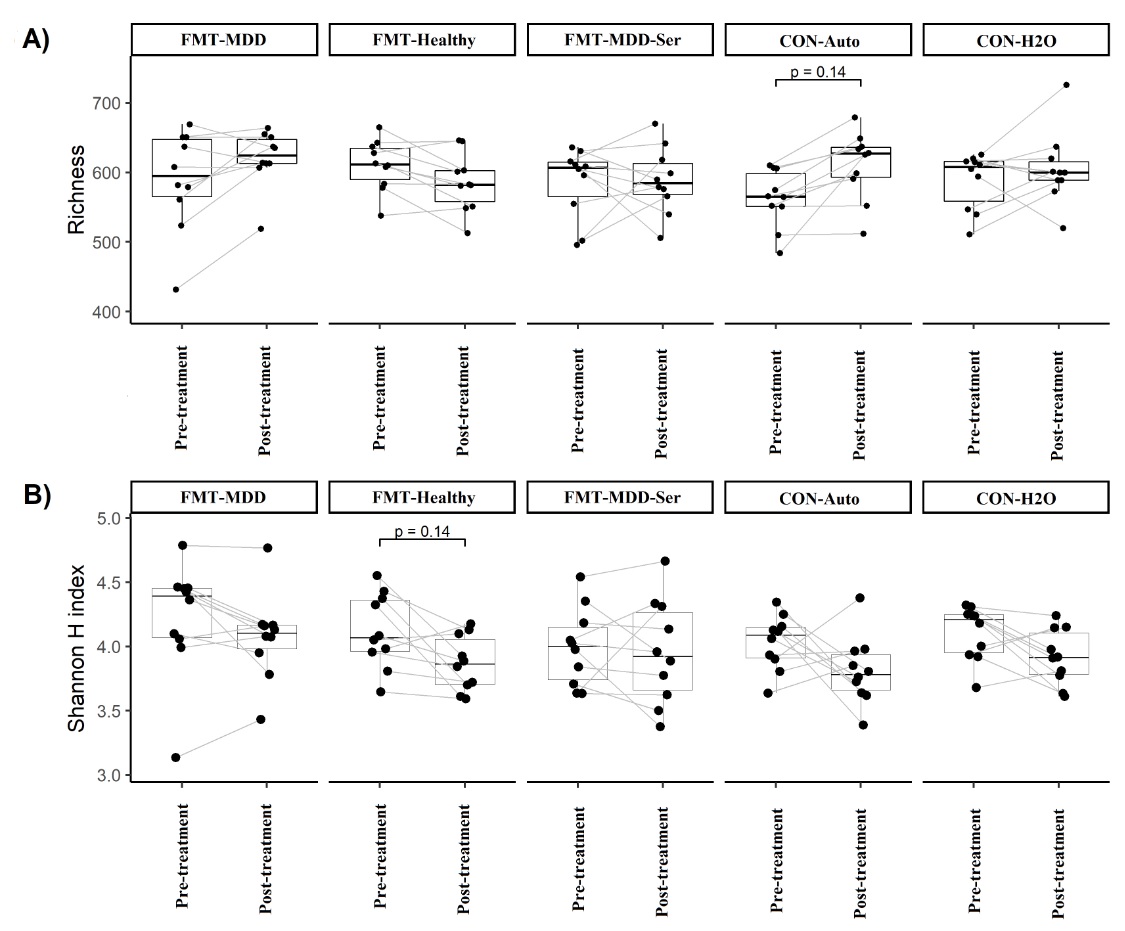

Supplement: Supplementary file 2 — Supplementary Figure 2. [file 41598_2021_1248_MOESM2_ESM.jpg]

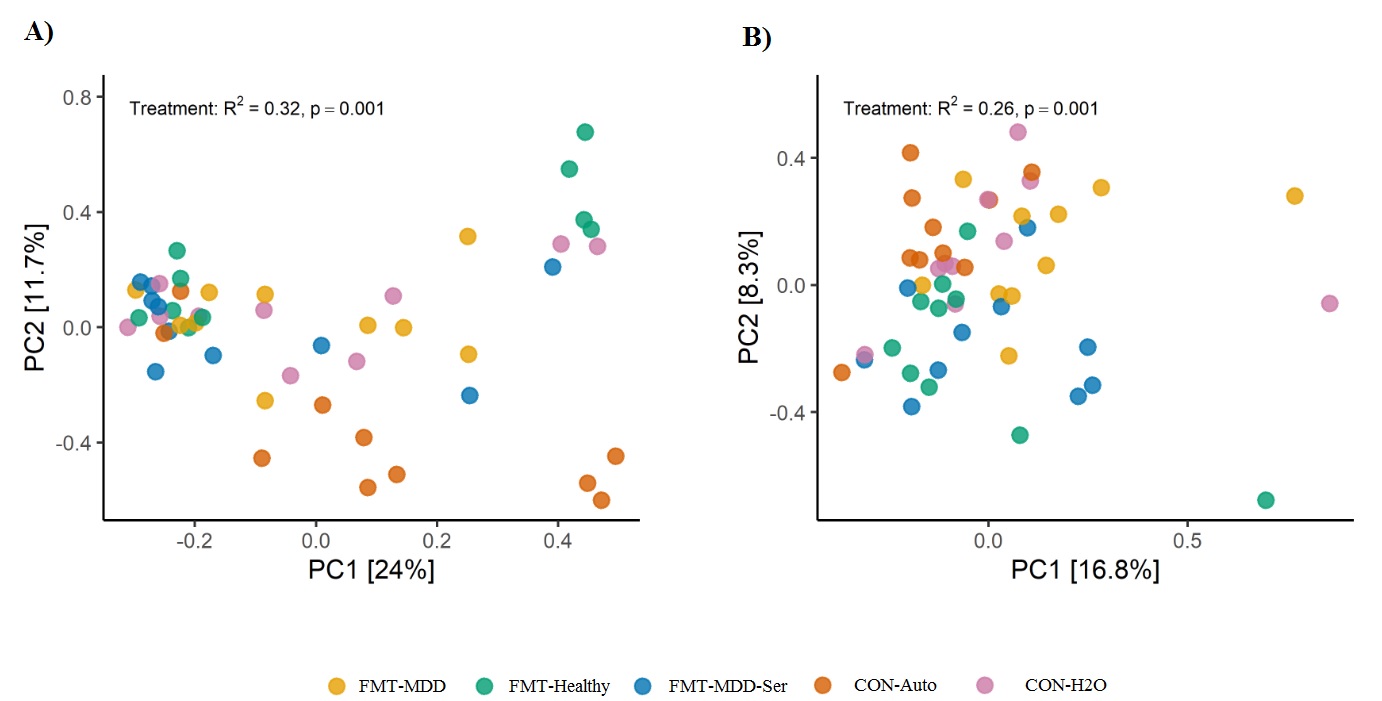

Supplement: Supplementary file 3 — Supplementary Figure 3. [file 41598_2021_1248_MOESM3_ESM.jpg]
