# Supplementary material for: Faecal microbiota transplantation from patients with depression or healthy individuals into rats modulates mood-related behaviour
Source: Sci Rep. 2021 Nov 8;11:21869. doi: 10.1038/s41598-021-01248-9 (PMC8575883; doi:10.1038/s41598-021-01248-9)
Supplement: Supplementary file 4 — Supplementary Legends. [file 41598_2021_1248_MOESM4_ESM.docx]

Supplementary Figure Legends

***Supplementary Figure 1* – Open field test for locomotor activity and rotarod test of time-to-exhaustion of FRL rats.** (A) Open field test determining the total distance travelled in 10 minutes. (B) Rotarod test measuring time-to-exhaustion, defined as the time from the animals was placed on a continuously accelerating cylinder until they fell off. P-values were included in the figure if they were below 0.2. Grey lines represent cohoused animals. FMT-MDD: Faecal microbiota transplantation from patients with MDD into rats. FMT-Healthy: Faecal microbiota transplantation from healthy individuals into rats. FMT-MDD-Ser: Faecal microbiota transplantation from patients with MDD into rats combined with treatment with sertraline. CON-Auto: Rats receiving auto-transplantations. CON-H2O: Rats receiving demineralised water.

**Supplementary Figure 2 – OTU richness and Shannon Index displaying the change in α-diversity from before to after interventions in FRL rats.** A) Box plots displaying the change in OTU richness. B) Box plots displaying the change in Shannon H Index. Pre-treatment; Faecal samples collected prior to initiation of the interventions. Post-treatment; Faecal samples collected after completion of the interventions. P-values were included in the figure if they were below 0.2. Grey lines symbolise samples from the same rat. FMT-MDD: Faecal microbiota transplantation from patients with MDD into rats. FMT-Healthy: Faecal microbiota transplantation from healthy individuals into rats. FMT-MDD-Ser: Faecal microbiota transplantation from patients with MDD into rats combined with treatment with sertraline. CON-Auto: Rats receiving auto-transplantations. CON-H2O: Rats receiving demineralised water.

**Supplementary Figure 3 – PCA plots of gut microbiota compositions before and after faecal microbiota transplantation in FRL rats.**  A) Separation of gut microbiota communities in rats prior to FMT based on their intervention (human versus non-human donor material recipients). B) Gut microbiota communities after FMT based on the donor material received. FMT-MDD: Faecal microbiota transplantation from patients with MDD into rats. FMT-Healthy: Faecal microbiota transplantation from healthy individuals into rats. FMT-MDD-Ser: Faecal microbiota transplantation from patients with MDD into rats combined with treatment with sertraline. CON-Auto: Rats receiving auto-transplantations. CON-H2O: Rats receiving demineralised water.
